# Assessment of Retinal Function Using Full-Field Electroretinography in Patients Undergoing Vitrectomy for a Macular Hole

**DOI:** 10.7759/cureus.97350

**Published:** 2025-11-20

**Authors:** Aleksandra Górska, Sebastian Sirek, Dorota Pojda-Wilczek

**Affiliations:** 1 Department of Ophthalmology, Faculty of Medical Sciences in Katowice, Kornel Gibiński University Clinical Centre, Medical University of Silesia, Katowice, POL

**Keywords:** erg, macular hole, phnr, photopic negative response, vitrectomy

## Abstract

Purpose

The aim of this study was to evaluate retinal function before and after vitrectomy in patients with a full-thickness macular hole (FTMH).

Methods

A total of 48 patients with FTMHs confirmed by spectral-domain optical coherence tomography (OCT) were enrolled. All underwent 23-gauge pars plana vitrectomy (PPV) with internal limiting membrane (ILM) peeling and gas tamponade. Retinal function was assessed using the RETeval™ (LKC Technologies, Inc., Germantown, MD) device before surgery and at one, three, six, and 12 months postoperatively. Electroretinography (ERG) recordings included standard photopic responses, photopic negative response (PhNR), and ON-OFF responses. Anatomical outcomes were evaluated using OCT.

Results

Statistical analysis was completed for 48 patients. Postoperative anatomical closure of the macular hole was achieved in all cases. A significant improvement was observed in both distance best corrected visual acuity (DBCVA) and near best corrected visual acuity (NBCVA) of the affected eye after surgery compared to preoperative values (p < 0.001). No significant differences were found between the ERG parameters of the affected eye and the healthy eye examined before surgery. Postoperatively, several photopic ERG implicit times (ITs) were prolonged in the operated eyes compared with the fellow eyes.

Conclusions

The RETeval™ system provides a non-invasive, standardized means of monitoring retinal function in patients with FTMHs undergoing vitrectomy. An FTMH appears to be a localized phenomenon, not associated with significant functional impairment of the entire retina, as evidenced by the lack of statistically significant differences in preoperative ERG parameters between the affected and fellow healthy eyes.

## Introduction

The assessment of retinal function in patients with macular holes undergoing vitrectomy is crucial for understanding both the structural and functional outcomes of surgical intervention. Full-field electroretinography (ERG) provides a non-invasive method to evaluate generalized retinal activity, offering insights into the integrity of photoreceptors, bipolar cells, and inner retinal layers. The RETeval™ (LKC Technologies, Inc., Germantown, MD), a portable ERG device, has emerged as a valuable tool for clinical electrophysiology, enabling standardized recordings even without pupillary dilation by adjusting stimulus luminance based on pupil size [1]. Our assessments adhered to International Society for Clinical Electrophysiology of Vision (ISCEV)-standardized protocols and incorporated specialized measurements like the photopic negative response (PhNR) and photopic ON-OFF ERG to gain a more detailed functional picture [2,3].

Macular holes, particularly idiopathic cases, involve progressive structural changes that impair central vision. While optical coherence tomography (OCT) provides detailed morphological data, functional assessments like ERG can detect early neuronal dysfunction before visible retinopathy develops [4]. The PhNR, a slow negative wave following the b-wave, is sensitive to inner retinal pathology, including ganglion cell dysfunction. Similarly, the photopic ON-OFF ERG helps differentiate ON- and OFF-pathway contributions, which may be affected in retinal disorders [3].

We hypothesized that a full-thickness macular hole (FTMH) would not affect generalized retinal function preoperatively, but subtle electrophysiological changes would occur post-vitrectomy. By analyzing ERG responses under photopic conditions, as well as specialized protocols, we seek to correlate functional outcomes with surgical results. The findings may refine patient selection for vitrectomy and improve postoperative monitoring, contributing to better clinical management of macular holes.

## Materials and methods

Of the 81 enrolled patients, 48 were included in the final analysis after excluding those with incomplete follow-up or postoperative complications (two patients had an FTMH in the fellow eye, while 12 did not attend the follow-up visit; five patients had amblyopia in the fellow eye; in 11 cases, the macular hole did not close postoperatively; and three patients developed retinal detachment). All patients diagnosed with an FTMH were scheduled to undergo pars plana vitrectomy (PPV). The study was conducted at the Department of Ophthalmology, University Clinical Center, Medical University of Silesia, Katowice, Poland, during the period between January 1, 2023, and June 30, 2025, and all patients provided informed consent. Each participant signed a written informed consent form before taking part in the study. Inclusion criteria comprised a clinical diagnosis of FTMHs confirmed by OCT, age >18 years, and willingness to comply with the study protocol. Exclusion criteria were categorized as ocular and systemic. Ocular exclusion criteria included a history of retinal surgery or other retinal pathologies, significant media opacities affecting image quality, uveitis or retinitis, ocular trauma, high myopia (≥−6.0 D sph), congenital or hereditary retinal disorders (i.e., age-related macular degeneration), other ocular diseases impairing retinal function (e.g., intraocular tumors or retinal vascular diseases), anterior segment abnormalities limiting pupil dilation, and prior intravitreal anti-vascular endothelial growth factor (anti-VEGF) injections. Systemic exclusion criteria comprised epilepsy, connective tissue disorders, ongoing oncological treatment, chronic immunosuppression, and current hyperbaric therapy. None of the patients had glaucoma.

All patients underwent standard 23-gauge PPV performed by an experienced retinal surgeon. The surgery involved inducing posterior vitreous detachment, peeling the internal limiting membrane (ILM) with standard inverted flap technique, brilliant blue staining, and gas tamponade using sulfur hexafluoride (SF6). Nineteen patients underwent combined PPV surgery with phacoemulsification cataract surgery and intraocular lens implantation. A Postoperative seven-day face-down positioning regimen was advised to facilitate macular hole closure [5].

Imaging and measurements

Distance visual acuity was measured using Snellen charts, and near visual acuity was assessed using Jaeger charts. Structural assessment of the retina was performed using Optovue, XR Avanti OCT (Visionix USA, Lombard, IL). This examination provided high-resolution cross-sectional images of the macula, allowing precise evaluation of macular hole dimensions, retinal layer integrity, and postoperative anatomical restoration.

Retinal function was assessed using the RETeval portable electrophysiological device (LKC Technologies, Inc.). Prior to testing, the pupil in the test eye was dilated with tropicamide hydrochloride 1.0%. A dedicated skin electrode array (Sensor Strip, LKC Technologies, Inc.) was positioned just below the lower eyelid margin, approximately 2 mm from its edge. The strip contained three integrated electrodes: the active (positive), the reference (negative), and the ground electrode, all mounted on a single adhesive element.

Light-adapted (LA) testing included two types of ERG to assess cone system function. The single-flash LA3 ERG consists of an a-wave, driven primarily by OFF-bipolar cells, and a b-wave, reflecting combined ON- and OFF-bipolar responses from L-, M-, and S-cones. The LA30 Hz flicker ERG, elicited by a flash near 30 Hz on an LA background, reflects mainly L- and M-cone-mediated ON- and OFF-bipolar cell activity, with minimal contribution from S-cones. Full-field ERGs sample the entire retina, with limited input from the macula [1].

Standardized ISCEV protocol for full-field photopic ERG was applied, along with specialized protocols including the PhNR and ON-OFF ERG [2,3].

Two types of visual stimulation were conducted for eliciting ON-OFF responses: a red flash on a green background (red/green, r/g) and a white flash on a white background (white/white, w/w). In the red/green approach, a red stimulus with a dominant wavelength of roughly 630 nm and a luminance of about 100 cd/m² is delivered against a constant green field (dominant wavelength around 530 nm, luminance near 50 cd/m²). The flash has a 100 ms duration, which enables a clear distinction between the ON and OFF response components. In the white/white configuration, a white flash of approximately 100 cd/m² is presented on a white background of around 50 cd/m², with matching spectral properties and the same 100 ms duration. For both paradigms, recordings are carried out following a minimum of 10 minutes of photopic light adaptation, as specified in the ISCEV protocol, to guarantee stable cone-driven retinal activity [3].

These protocols were selected to evaluate generalized retinal function as well as specific inner retinal pathway integrity. The order of tests was: LA3.0, LA30, PhNR, red/green, and white/white [6].

The analysis encompassed the peak time (implicit time, IT) and amplitude (A) of both the a-wave and b-wave in the LA3 ERG, as well as the ON response. For the LA30 Hz ERG, the IT and amplitude were analyzed. Regarding the PhNR, its amplitude and the W-ratio were analyzed. The W-ratio was included in the examination report, along with the other parameters subjected to analysis. This ratio is calculated automatically and is presented in the examination report.

The W-ratio was defined as (b - pmin)/(b - a), where “b” represents the b-wave amplitude from the baseline, “pmin” is the minimum amplitude of the negative trough (from the baseline), and “a” is the a-wave amplitude from the baseline. This W-ratio represents the inverse of the photopic response described by Mortlock et al. [7]. This ratio reflects the relative depth of the PhNR response and helps to standardize measurements across individuals [7,8].

Patients were examined before surgery and at one, three, six, and 12 months after surgery. 

Ethical considerations

The study was conducted in accordance with the principles outlined in the Declaration of Helsinki and received approval from the Bioethics Committee (nr. PCN/CBN/0052/KB1/122/22).

Written informed consent was obtained from all participants prior to their enrollment. 

Statistical analysis

The Shapiro-Wilk test and quantile-quantile plots were used to assess the normality of the distributions of the analyzed data.

In order to compare the data of two independent groups with normal or skewed distributions, the Student’s t-test or the Mann-Whitney test was performed, respectively. In turn, for two dependent groups with a skewed distribution, the Wilcoxon signed-rank test was used. The homogeneity of variance was assessed using the Fisher-Snedecor and Levene tests (ANOVA). For groups with unequal variances, Welch’s t-test with independent variance estimation was used. The comparison of data from multiple dependent samples with a normal distribution and preserved sphericity of results was performed using ANOVA for repeated measures. Sphericity (homogeneity of variance) was tested using Mauchly’s test. In the absence of preserved sphericity of the data, the Greenhouse-Geisser correction was used in the ANOVA test to assess the differences. In turn, the analysis for multiple dependent samples with a skewed distribution was performed using Friedman’s ANOVA test. For statistically significant results, detailed comparisons were made using contrast analysis.

Spearman’s rank correlation test was used to assess the relationship and determine the strength of the relationship between two skew-distributed variables.

The analysis of the relationship between qualitative characteristics was performed using the Chi-squared test, with Yates’ correction applied for small expected frequencies.

A p-value of <0.05 was considered statistically significant, and all tests were two-tailed.

Statistical analysis and figures were performed using Statistica version 13 (TIBCO Software Inc., 2017).

## Results

Forty-eight patients participated in the study, of whom 37 (77%) were women and 11 (23%) were men. The mean age with standard deviation for the entire group was 66.8 ± 5.6 years. No significant differences in age were found between the sexes (p = 0.91). Specialized examinations of the affected eye (FTMH) were performed before surgery (at t0) and after one, three, six, and 12 months (at t1, t3, t6, and t12, respectively). Examinations of the healthy eye (as a control group) were performed before surgery and 12 months after surgery.

Based on tests conducted before surgery (at t0), significant differences in distance best corrected visual acuity (DBCVA) and near best corrected visual acuity (NBCVA) [logMAR] were found between the FTMH eye group and the healthy fellow eye group (control group), respectively (p < 0.001 and p < 0.001) (Table 1). The DBCVA [logMAR] values in the affected eye group were higher than the DBCVA [logMAR] values in the control group, indicating poorer distance visual acuity in the affected eye compared to the healthy eye. In turn, the NBCVA [logMAR] values in the affected eye group were lower than the NBCVA [logMAR] values in the control group, indicating poorer near visual acuity in the affected eye compared to the healthy eye. Follow-up examinations performed 12 months after surgery (t12) also showed significant differences in DBCVA and NBCVA [logMAR] of the affected eye compared to the healthy eye, respectively (p < 0.001 and p < 0.05). In addition, a significant difference was found between the DBCVA and NBCVA values of the affected eye before and after surgery (p(t0 vs. t12) < 0.001), indicating a significant improvement in distance and near visual acuity as a result of the surgery. Detailed data are presented in Table 1.

**Table 1:** DBCVA and NBCVA of preoperative (t0) and postoperative (t12) eyes with FTMH (FTMH group) and fellow eyes (control group) *The Mann-Whitney test for independent samples. #The Wilcoxon signed-rank test for dependent samples. Me (Q1-Q2), median (lower-upper quartile); p < 0.05, statistical significance; DBCVA, distance best corrected visual acuity; NBCVA, near best corrected visual acuity; FTMH, full-thickness macular hole

n = 48	Timepoint	FTMH group	Control group	p-value
DBCVA [logMAR]	t_0_	1.097 (1.00 to 1.398)	0.000 (0.000 to 0.046)	<0.001*
	t_12_	0.398 (0.222 to 0.699)	0.000 (0.000 to 0.046)	<0.001*
	p(t_0_ vs. t_12_)	<0.001#	1.00#	-
NBCVA [logMAR]	t_0_	-0.352 (-0.477 to -0.176)	0.301 (0.301 to 0.301)	<0.001*
	t_12_	0.301 (0.213 to 0.301)	0.301 (0.301 to 0.301)	<0.05*
	p(t_0_ vs. t_12_)	<0.001#	1.00#	-

A statistically significant difference was demonstrated between the DBCVA [logMAR] values in the FTMH group examined at one, three, six, and 12 months after surgery (p < 0.001). The median DBCVA [logMAR] in the FTMH eye group before surgery was 1.097 (see Table 1), and at three months after surgery, it decreased to 0.398, maintaining this DBCVA value in subsequent follow-up examinations (six and 12 months after surgery). A detailed analysis of changes in this parameter between follow-up examinations t3 vs. t6 and t6 vs. t12 indicated significant changes in DBCVA up to six months after surgery (p < 0.01) and no significant changes thereafter (p = 0.87).

With regard to the analysis of NBCVA test results for the affected eye, significant changes were also demonstrated in subsequent follow-up examinations after surgery (p < 0.001). The median NBCVA [logMAR] in the affected eye group before surgery was −0.352 (see Table 1), and at three months after surgery, it increased to 0.301, maintaining this value in subsequent follow-up examinations (six and 12 months after surgery). A detailed analysis of changes in this parameter at six and 12 months after surgery (t6 vs. t12) showed significant changes in NBCVA values even up to 12 months after surgery (p < 0.05). Detailed data for the DBCVA and NBCVA parameters in [logMAR] are presented in Table 2.

**Table 2:** DBCVA and NBCVA of eyes with macular hole (MH group) at different times of postoperative check-up visits (after 1, 3, 6, and 12 months: t1, t3, t6, and t12, respectively) The Friedman rank test Me (Q1-Q2), median (lower-upper quartile); p < 0.05, statistical significance; DBCVA, distance best corrected visual acuity; NBCVA, near best corrected visual acuity; FTMH, full-thickness macular hole

n = 48	Timepoint (postoperative)			
	FTMH group (t_1_)	FTMH group (t_3_)	FTMH group (t_6_)	FTMH group (t_12_)
DBCVA [logMAR]	0.523 (0.398 to 1.000)	0.398 (0.301 to 0.699)	0.398 (0.222 to 0.611)	0.398 (0.222 to 0.699)
NBCVA [logMAR]	0.000 (-0.176 to 0.301)	0.301 (0.000 to 0.301)	0.301 (0.063 to 0.301)	0.301 (0.213 to 0.301)

Postoperative DBCVA values in the affected eye group were characterized by a statistically significant, high positive correlation with the minimum and maximum FTMH diameter (p < 0.001), both at one month and 12 months after surgery.

Postoperative NBCVA values in the affected eye group were characterized by a statistically significant, high, and moderate positive correlation with the minimum and maximum FTMH diameter (p < 0.001 and p < 0.05), both at one month and 12 months after surgery.

However, postoperative DBCVA and NBCVA [logMAR] values of the affected eye increased and decreased, respectively, in relation to the increasing minimum and maximum FTMH diameters, indicating that poorer distance and near visual acuity were associated with the larger FTMH diameters of the affected eye determined before surgery.

No statistically significant correlation was found between the duration of FTMH before surgery and the minimum (rs = 0.26, p = 0.077) and maximum (rs = 0.25, p = 0.083) FTMH diameter.

No significant differences were found between the ERG parameters of the affected eye and the healthy eye examined before surgery. The presence of FTMH, therefore, does not significantly affect the values of the parameters examined in relation to the healthy eye. Detailed data are summarized in Table 3.

**Table 3:** Comparison of ERG parameters of the FTMH eye with the fellow eye collected before surgery (at t0) *The Mann-Whitney test for independent samples. #The Student’s t-test for independent samples. MSD, mean standard deviation; ERG, electroretinography; FTMH, full-thickness macular hole; Me (Q1-Q3), median (lower-upper quartile); LA3, light-adapted; LA30, light-adapted 30 Hz; a, a-wave; b, b-wave; IT, implicit time (ms); A, amplitude (uV); PhNR, photopic negative response; r/g, red/green; w/w, white/white; p < 0.05, statistical significance

Lp.	ERG	FTMH eye	Fellow eye	p-value
1	LA3 a IT	12.80 (11.50 to 13.80)	13.00 (12.40 to 13.50)	0.342*
2	LA3 a A	5.691.98	6.052.10	0.392#
3	LA3 b IT	29.941.64	29.941.94	1.000#
4	LA3 b A	24.767.55	25.957.61	0.444#
5	LA30 IT	26.10 (25.40 to 27.45)	26.00 (25.40 to 27.05)	0.829*
6	LA30 A	20.016.44	21.695.84	0.186#
7	PhNR A	4.55 (3.35 to 5.50)	4.75 (3.65 to 6.05)	0.358#
8	W-ratio	0.97 (0.88 to 1.12)	0.97 (0.91 to 1.09)	0.968#
9	r/g ON IT	39.15 (34.45 to 42.30)	40.30 (38.05 to 41.60)	0.337*
10	r/g ON A	13.954.52	14.114.08	0.854#
11	w/w ON IT	36.452.14	36.462.14	0.977#
12	w/w ON A	15.214.24	16.184.13	0.258#

Significant differences were demonstrated for postoperative ERG parameters: LA3 a-wave IT (p < 0.01), LA3 b-wave IT (p < 0.01), LA30 IT (p < 0.05), and white/white (w/w) ON IT (p < 0.001) in the affected eye compared to the healthy eye. The surgery, therefore, contributed significantly to the change in the above four parameters determining the function of the MH retina compared to the healthy eye. Detailed data are summarized in Table 4.

**Table 4:** Comparison of ERG parameters of the FTMH eye with the fellow eye collected 12 months after surgery (at t12) *The Mann-Whitney test for independent samples. #The Student’s t-test for independent samples. MSD, mean standard deviation; ERG, electroretinography; FTMH, full-thickness macular hole; Me (Q1-Q3), median (lower-upper quartile); LA3, light-adapted; LA30, light-adapted 30 Hz; a, a-wave; b, b-wave; IT, implicit time (ms); A, amplitude (uV); PhNR, photopic negative response; r/g, red/green; w/w, white/white; p < 0.05, statistical significance

Lp.	ERG	FTMH eye	Control group	p-value
1	LA3 a IT	13.15 (12.25 to 14.35)	12.60 (11.65 to 13.50)	<0.01#
2	LA3 a A	4.60 (3.40 to 6.25)	5.20 (4.15 to 6.40)	0.441*
3	LA3 b IT	30.80 (30.10 to 32.40)	30.25 (29.30 to 30.85)	<0.01#
4	LA3 b A	21.615.79	23.387.50	0.198*
5	LA30 IT	26.95 (26.05 to 28.15)	26.20 (25.55 to 27.20)	<0.05#
6	LA30 A	17.095.04	19.015.41	0.076*
7	PhNR A	4.50 (3.50 to 6.65)	4.50 (3.35 to 6.35)	0.947#
8	W-ratio	1.13 (1.06 to 1.20)	1.10 (1.05 to 1.21)	0.276#
9	r/g ON IT	40.663.75	39.823.79	0.277*
10	r/g ON A	14.75 (11.20 to 17.35)	13.55 (11.75 to 17.20)	0.475#
11	w/w ON IT	38.20 (37.20 to 39.30)	36.75 (35.75 to 38.00)	<0.001#
12	w/w ON A	14.30 (11.15 to 17.60)	14.55 (12.20 to 17.75)	0.650#

Next, a detailed analysis of changes in ERG parameters following surgery was performed, comparing the results of tests collected before surgery and during follow-up examinations at one, three, six, and 12 months after surgery.

Significant changes in the ERG parameters of the affected eye were demonstrated: LA3 a-wave IT (p < 0.05), LA3 b-wave IT (p < 0.001), LA3 b-wave A (p < 0.01), LA30 IT (p < 0.001), LA30 A (p < 0.001), W-ratio (p < 0.001), r/g ON IT (p < 0.01), r/g ON A (p < 0.001), w/w ON IT (p < 0.001), and w/w ON A as a result of surgery. A detailed comparative analysis of ERG parameters over time showed significant changes in LA3 b-wave IT, W-ratio, w/w ON IT up to 12 months, LA3 a-wave IT, LA3 b-wave A, LA30 IT, LA30 A, w/w ON A up to six months, r/g ON IT, and r/g ON A up to one month after surgery. Detailed data are presented in Table 5.

**Table 5:** Comparison of the ERG parameters in the eye group with FTMH at different times: preoperative (t0) and postoperative check-up visits (after 1, 3, 6, and 12 months: t1, t3, t6, and t12, respectively) *The Friedman rank test for dependent samples. #The one-way ANOVA test for independent samples. MSD, mean standard deviation; ERG, electroretinography; FTMH, full-thickness macular hole; Me (Q1-Q3), median (lower-upper quartile); LA3, light-adapted; LA30, light-adapted 30 Hz; a, a-wave; b, b-wave; IT, implicit time (ms); A, amplitude (uV); PhNR, photopic negative response; r/g, red/green; w/w, white/white; p < 0.05, statistical significance

n = 48	Timepoint (postoperative)					p-value
	FTMH group (t0)	FTMH group (t1)	FTMH group (t3)	FTMH group (t6)	FTMH group (t12)	
LA3 a IT	12.80 (11.50 to 13.80)	13.75 (12.45 to 14.65)	13.40 (12.40 to 14.15)	13.15 (12.35 to 14.10)	13.15 (12.25 to 14.35)	<0.05*
LA3 a A	5.50 (4.40 to 7.05)	4.80 (3.85 to 6.55)	5.25 (3.85 to 6.50)	5.15 (4.05 to 6.50)	4.60 (3.40 to 6.25)	0.455*
LA3 b IT	29,941,64	31.65 1.91	31.36 1.82	30.83 1.55	31.11 1.75	<0.001#
LA3 b A	24.767.55	23.626.32	23.186.80	21.514.96	21.615.79	<0.01#
LA30 IT	26.10 (25.40 to 27.45)	27.45 (26.35 to 29.35)	27.20 (26.26 to 28.70)	27.20 (26.15 to 28.15)	26.95 (26.05 to 28.15)	<0.001*
LA30 A	20.016.44	18.075.31	17.885.69	16.734.50	17.095.04	<0.001#
PhNR A	4.55 (3.35 to 5.50)	4.20 (3.50 to 5.30)	4.60 (3.40 to 5.80)	4.50 (3.50 to 5.45)	4.50 (3.50 to 6.65)	0.181*
W-ratio	0.97 (0.88 to 1.12)	0.97(0.89 to 1.09)	1.02 (0.89 to 1.15)	1.09 (0.98 to 1.20)	1.13 (1.06 to 1.20)	<0.001*
r/g ON IT	39.15 (34.45 to 42.30)	40.80 (38.75 to 43.80)	41.95 (40.75 to 43.40)	41.30 (38.80 to 43.70)	41.50 (38.30 to 43.50)	<0.01*
r/g ON A	13.954.52	16.154.94	15.635.14	14.884.58	14.804.78	<0.001#
w/w ON IT	36,452,14	38.942.89	37.912.35	37.452.72	38.032.24	<0.001#
w/w ON A	15.214.24	16.374.62	15.784.79	14.683.99	14.624.62	<0.05#

It has been shown that among the ERG parameters studied, only the W ratio correlates significantly on average and negatively with the waiting time for surgery (rs=-0.34, p<0.05), which means that W ratio values decrease on average but significantly with increasing waiting time for surgery (Table 6).

**Table 6:** Correlation of postoperative ERG parameters (at t12) with FTMH duration to PPV The Spearman rank test ERG, electroretinography; FTMH, full-thickness macular hole; PPV, pars plana vitrectomy; rs, Spearman correlation coefficient; LA3, light-adapted; LA30, light-adapted 30 Hz; a, a-wave; b, b-wave; IT, implicit time (ms); A, amplitude (uV); PhNR, photopic negative response; r/g, red/green; w/w, white/white; p < 0.05, statistical significance

ERG (t_12_)	Time to PPV	
	r_s_	p-value
LA3 a IT (ms)	-0.09	0.545
LA3 a A (mV)	0.1	0.483
LA3 b IT (ms)	0.06	0.668
LA3 b A (mV)	-0.03	0.859
LA30 IT (ms)	0.11	0.476
LA30 A (mV)	0.01	0.933
PhNR A (mV)	-0.26	0.07
W-ratio	-0.34	<0.05
r/g ON IT (ms)	0.18	0.225
r/g ON A (mV)	0.05	0.756
w/w ON IT (ms)	0.06	0.705
w/w ON A (mV)	0.02	0.883

No statistically significant correlation was found between improvement in DBCVA of the FTMH eye following surgery and patient age (divided into groups: below and above the median age = 67.5 years; p = 1.00).

No statistically significant correlation was found between changes in the FTMH eye ratio following surgery and patient age (divided into groups: below and above the median age = 67.5 years; p = 0.745).

A statistically significant difference in the ERG IT parameter was demonstrated: LA3 a-wave and IT (p < 0.05) and LA30 IT (p < 0.05) between the two groups of patients defined as above and below the median age (67.5 years). No significant difference was found between the studied patient groups for the remaining ERG parameters: IT and W-ratio.

No significant differences were found for ERG and DBCVA parameters between the two groups of patients: those undergoing combined surgery (phacoemulsification with PPV) and those undergoing PPV. Therefore, combined surgery does not significantly affect the differences in the above-mentioned characteristics in relation to the group of patients undergoing only one procedure (PPV).

## Discussion

This longitudinal study offers robust electrophysiological evidence that an FTMH constitutes a localized macular pathology rather than a manifestation of generalized retinal dysfunction. The absence of statistically significant differences in preoperative full-field ERG parameters, including standard photopic responses, the PhNR, and ON-OFF pathway responses, between the affected and fellow eyes represents a key finding. These results indicate that the functional integrity of the global retina, including its inner retinal layers as reflected by the PhNR, remains preserved outside the foveal lesion. Accordingly, the present data suggest that preoperative full-field ERG does not contribute meaningfully to the qualification process for PPV, as it fails to reveal evidence of widespread retinal impairment in this patient group.

The most notable observations emerged in the postoperative period. A consistent prolongation of ITs was recorded across major photopic ERG components, including the LA3 a-wave, LA3 b-wave, LA30 response, and the b-wave of the ON-response (w/w ON IT). These temporal delays were evident at the earliest postoperative assessment and persisted for up to 12 months, despite a concurrent and statistically significant improvement in visual acuity. This divergence between restored high-contrast visual acuity and enduring electrophysiological abnormalities highlights the sensitivity of ERG in detecting subtle, subclinical alterations in retinal function that remain undetectable through standard visual acuity testing.

Our observations resonate with the earlier findings of Ueno et al., who note a decrease in the PhNR wave after macular hole surgery [9]. Although our study did not see a reduction in the amplitude of that specific wave, the delays we found in the timing of multiple other pathways suggest a wider, though temporary, disruption in how the retina processes light signals following the operation [9].

The causative factor for these functional changes appears to be the surgical procedure itself. The combination of PPV, brilliant blue staining, ILM peeling, and intraocular gas tamponade creates a perfect storm of mechanical, biochemical, and metabolic challenges for the retina. The peeling of the ILM, a crucial basement membrane for Müller cell end-feet, inevitably causes trauma to these glial cells. Müller cells play a vital role in retinal potassium siphoning, neurotransmitter recycling, and overall retinal homeostasis. Their disruption can directly lead to altered potassium kinetics, which is a key determinant of the b-wave IT, potentially explaining the increased IT we observed [10].

An encouraging finding from our longitudinal data is the transient nature of this surgically induced dysfunction. For several parameters, such as the LA3 a-wave IT and the LA30 amplitude, the most significant changes were observed at the one- and three-month control, with a trend toward stabilization or partial recovery by the six- and 12-month time points. This pattern of gradual functional recovery aligns with the findings of Machida et al., who reported a progressive increase in focal macular electroretinograms (fmERG) amplitudes over a 12-month period following FTMH surgery, with the PhNR showing a particularly robust recovery [11]. This indicates a remarkable capacity for neural repair and functional plasticity in the macular region following successful anatomical closure. The restoration of the foveal microstructure likely allows for the re-establishment of more normal synaptic connections and Müller cell function, facilitating this slow recovery process [11].

An important conceptual framework for understanding our results is provided by the work of Machida et al. on the differences between glaucoma and post-MH ganglion cell complex (GCC) thinning [11]. They found that for a similar degree of GCC thinning, the functional loss (as measured by the PhNR) was significantly greater in glaucoma patients than in post-vitrectomy MH patients. This suggests that the pathophysiology of RGC dysfunction following surgical trauma is fundamentally different from the progressive neurodegeneration in glaucoma. In the postoperative state, the thinning may reflect a combination of mechanical rearrangement, transient atrophy, and axonal disruption, which has a different functional consequence than the chronic, progressive apoptosis seen in glaucoma. Importantly, none of our patients had glaucoma, which supports the interpretation that any observed ERG changes reflect post-surgical effects rather than glaucomatous damage. This framework helps explain why our patients could exhibit ERG alterations while still achieving good visual acuity recovery [12].

The W-ratio, which reflects the function of retinal ganglion cells (RGC) relative to the activity of the outer retinal layers, was found to be higher than initially expected in our cohort. This suggests that RGC function remains largely preserved, while the functional impairment primarily affects the outer retinal layers. The surgical procedure induces transient retinal dysfunction, with the most pronounced disturbances observed in the early postoperative period. These findings indicate that the ERG alterations observed shortly after surgery are largely functional rather than indicative of permanent RGC loss, highlighting the resilience of RGCs compared to the more vulnerable outer retinal structures. The findings of this study indicate that, among all analyzed ERG parameters, the W-ratio was the only one showing a significant association with the interval between the onset of FTMH and the performed PPV. Specifically, longer delays were associated with a reduction in W-ratio values, which points to a gradual decline in the function of the inner retinal layers. As these layers are especially susceptible to ischemic damage, extended waiting periods may contribute to their progressive dysfunction. This finding emphasizes the clinical relevance of early surgical intervention for maintaining retinal function and suggests that unnecessary postponement of treatment may have a negative impact on long-term visual outcomes.

The findings of this study, considered alongside existing literature, refine the current understanding of retinal function in patients with FTMHs undergoing vitrectomy. The results reinforce the concept that the macular hole represents a localized structural defect rather than an indicator of generalized retinal dysfunction. Nevertheless, surgical closure of the hole, although highly effective in achieving anatomical repair and functional recovery of central vision, is associated with a subtle yet measurable disturbance in global retinal physiology, manifested primarily as a transient delay in photopic response timing.

The use of the RETeval™ system enabled the detection of these nuanced electrophysiological alterations in a non-invasive and reproducible manner, underscoring its potential utility in postoperative functional monitoring. Future investigations correlating extended ERG parameters with patient-reported visual outcomes and microperimetric data may provide deeper insight into the clinical significance of these electrophysiological findings. Moreover, ongoing developments in surgical methodology, such as the optimization of intraoperative dyes and strategies aimed at modulating Müller cell reactivity, hold promise for further reducing the subtle functional consequences that may accompany anatomical restoration.

## Conclusions

The results of this study do not support the hypothesis that an FTMH is part of a generalized retinal dysfunction. Instead, an FTMH appears to be a localized phenomenon, not associated with significant functional impairment of the entire retina, as evidenced by the lack of statistically significant differences in preoperative ERG parameters between the affected and fellow healthy eyes. Consequently, preoperative ERG does not appear to be useful for qualifying patients with FTMH for PPV.

However, the postoperative ERG findings indicate that the surgical procedure itself transiently disturbs photopic retinal function. Although this dysfunction diminishes over time, the ERG parameters did not fully return to baseline levels within the 12-month observation period. This suggests that the surgical intervention, rather than the macular hole alone, temporarily alters retinal physiology. Therefore, it must be acknowledged that any vitreoretinal surgery may have a measurable, though often transient, impact on retinal function.
